# Supplementary material for: HIVprotI: an integrated web based platform for prediction and design of HIV proteins inhibitors
Source: J Cheminform. 2018 Mar 9;10:12. doi: 10.1186/s13321-018-0266-y (PMC5845081; doi:10.1186/s13321-018-0266-y)
Supplement: Supplementary file 2 — Additional file 2. Source code of HIVProtI web server. [file 13321_2018_266_MOESM2_ESM.zip › HIVprotI_Source-code/hivprotI/jsme/JSME.html]

JSME demo page


Your web browser must have JavaScript enabled in order for this
application to display correctly.

# JSME demo page

|  |  |
| --- | --- |
| Set options: | |
| reaction,noquery,oldlook | Set options |

Import predefined
chemical structure to applet:

Read JME
String

|  |  |  |
| --- | --- | --- |
| Export chemical structure from applet: | | |
| Get molfile | Get smiles | Get JME string |
|  | | |

See also description
of the JME API.
